# Outcomes of a Risk-Stratified Protocol for Preventing Peristomal Skin Complications in Patients with an Ostomy: A Cohort Study

**DOI:** 10.3390/nursrep15050179

**Published:** 2025-05-20

**Authors:** Francesco Carlo Denti, Eliana Guerra, Francesca Caroppo, Pietro Abruzzese, Fabrizio Alessi, Filippo Barone, Pasqualina Bernardino, Massimiliano Bergamini, Maria Cristina Bernardo, Gloria Bosio, Paula Carp, Manuela Cecconello, Annalinda Cerchier, Francesca Croci, Rita Detti, Mina Milenova Dimitrova, Cristina Di Pasquale, Maria Rosaria D’Ippolito, Simona Ditta, Erica Ducci, Anna Belloni Fortina, Stefano Frascarelli, Marianna Galante, Rita Guarino, Nicola Leggio, Elisabetta Livio, Alessandra Marchetti, Francesca Marelli, Rita Mastropaolo, Viviana Melis, Nicola Palmiero, Arianna Panarelli, Anna Lea Pascali, Francesco Pizzarelli, Laura Precisi, Cinzia Rastello, Silvia Regaglia, Rossana Elvira Rinaldi, Nadia Rumbolo, Claudio Sansone, Angela Santelli, Giovanni Sarritzu, Stefano Sfondrini, Sara Stanzani, Mattia Stella, Margherita Walterova, Rosario Caruso

**Affiliations:** 1 Stomacare Service, IRCCS San Raffaele Institute, 20132 Milan, Italy; 2 Enterostomal Rehabilitation Clinic, ASST Spedali Civili Brescia, 25123 Brescia, Italy; 3 Dermatology Unit, Department of Medicine DIMED, University of Padua, 35131 Padua, Italy; 4 Department of Womens’ and Children’s Health (SDB), University of Padova, 35131 Padua, Italy; 5 Stomacare Service, Ospedale Bellaria Carlo Alberto Pizzardi, 40139 Bologna, Italy; 6 Stomacare Service, Ospedale di Legnano, ASST Ovest Milanese, 20025 Legnano, Italy; 7 Stomacare Service, Ospedale Generale Regionale Francesco Miulli, 70021 Acquaviva delle Fonti, Italy; 8 Stomacare Service, A.O.O.R Villa Sofia-V. Cervello, 90146 Palermo, Italy; 9 Stomacare Service, Presidio Ospedaliero Universitario “Santa Maria della Misericordia”, 45100 Rovigo, Italy; 10 Stomacare Service, Presidio Ospedaliero di Ivrea, ASL Torino 4, 10015 Ivrea, Italy; 11 Stomacare Service, Ospedale di Rivoli, 10098 Rivoli, Italy; 12 Stomacare Service, Presidio Ospedaliero Martini, 10141 Torino, Italy; 13 Stomacare Service, Ospedale di Feltre, 32032 Feltre, Italy; 14 Stomacare Service, Ospedale di San Donà di Piave, 30027 San Donà di Piave, Italy; 15 Stomacare Service, Ospedale “Val Vibrata” di Sant’Omero, ASL Teramo, 64027 Teramo, Italy; 16 Stomacare Service, Azienda Ospedaliero, Universitaria Senese, 53100 Siena, Italy; 17 Stoma Therapy Service, P. Pederzoli Hospital, 37019 Peschiera del Garda, Italy; 18 Stomal Therapy Outpatient Service, European Institute of Oncology IRCCS, 20141 Milan, Italy; 19 Stomacare Service, Azienda Ospedaliera di Rilievo Nazionale Antonio Cardarelli, 80131 Napoli, Italy; 20 Stomacare Service, Ospedale Luigi Sacco, 20157 Milan, Italy; 21 Stomacare Service, USL Umbria 1, 06127 Perugia, Italy; 22 Stomacare Service, Ospedale Santa Chiara, 38123 Trento, Italy; 23 Stomacare Service, IRCCS Fondazione G. Pascale di Napoli, 80131 Napoli, Italy; 24 Stomacare Service, Fondazione IRCCS Ca’ Granda Ospedale Maggiore Policlinico, 20122 Milan, Italy; 25 Stomacare Service, Clinica Ospedaliero, Universitaria Policlinico Umberto I, 00161 Rome, Italy; 26 Stomacare Service, Fondazione IRCCS Istituto Nazionale dei Tumori, 20133 Milan, Italy; 27 Stomacare Service, Ospedale di Bolzano, 39100 Bolzano, Italy; 28 Stomacare Service, ASST Papa Giovanni XXIII, 24127 Bergamo, Italy; 29 Stomacare Service, Policlinico di Bari Ospedale “Giovanni XXIII”, 70124 Bari, Italy; 30 Stomacare Service, Asl Lecce, 73100 Lecce, Italy; 31 Stomacare Service, Azienda Ospedaliera di Padova, 35131 Padua, Italy; 32 Stomacare Service, Azienda Ospedaliero Universitaria Pisana, 56126 Pisa, Italy; 33 Stomacare Service, Azienda Ospedaliera Universitaria San Luigi Gonzaga, 10043 Orbassano, Italy; 34 Stomacare Service, Ospedale Civile Santissima Annunziata, 07100 Sassari, Italy; 35 Stomacare Service, Ospedale Pesenti Fenaroli, 24022 Alzano Lombardo, Italy; 36 Stomacare Service, Fondazione IRCCS San Gerardo dei Tintori, 20900 Monza, Italy; 37 Stomacare Service, Azienda Ospedaliera San Giovanni Addolorata, 00184 Rome, Italy; 38 Stomacare Service, Ospedale Pio XI, 20832 Desio, Italy; 39 Stomacare Service, Policlinico Universitario Monserrato “Duilio Casula”, 09042 Monserrato, Italy; 40 Stomacare Service, Ospedale S. Anna, ASST Lariana, 22042 Como, Italy; 41 Stomacare Service, Ospedale Santa Maria delle Croci, 48121 Ravenna, Italy; 42 Stomacare Service, IRCCS Saverio De Bellis, 70013 Castellana Grotte, Italy; 43 Health Professions Research and Development Unit, IRCCS Policlinico San Donato, 20097 San Donato Milanese, Italy; 44 Department of Biomedical Sciences for Health, University of Milan, 20133 Milan, Italy

**Keywords:** peristomal skin complications, ostomy care, risk-stratified education, self-care, health-related quality of life, patient satisfaction, Poisson regression, stoma care outcomes, risk management

## Abstract

Background/Objectives: Peristomal skin complications (PSCs) are common among patients with ostomies, significantly impacting quality of life and increasing healthcare utilization. This study aimed to evaluate the effects of the Dermamecum protocol, a risk-stratified educational intervention, on the prevention of PSCs, self-care improvements, health-related quality of life (HRQoL), and patient satisfaction over a 90-day follow-up period. Methods: This prospective cohort study included 305 patients stratified into three risk-based groups (green, yellow, and red paths) according to the Dermamecum protocol. Primary outcomes included PSC rates at 30, 60, and 90 days. Secondary outcomes included self-care scores, HRQoL, and patient satisfaction. Comparative analyses and trend assessments were performed across groups and time points. Temporal trends in PSCs were analyzed using Poisson regression. Results: Early PSC rates were 8.5% at 30 days, with late complications at 7.9% and 6.2% at 60 and 90 days, respectively. No significant differences in PSC rates were observed between paths. Self-care scores improved over time, with stability across groups and domains. HRQoL remained stable, with minor fluctuations in physical and mental components. Patient satisfaction was high across all paths. Poisson regression identified significant temporal trends in PSC rates, with higher risks at 30, 60, and 90 days compared to baseline. Age, BMI, and path assignment (lower risk for the green path) were significant predictors of PSCs. Conclusions: The Dermamecum protocol effectively maintained low PSC rates, supported self-care, and sustained HRQoL and patient satisfaction. These findings highlight the value of risk-stratified, patient-centered interventions in ostomy care. Further studies are needed to validate these results and explore long-term outcomes.

## 1. Introduction

Peristomal skin complications (PSCs) are among the most frequent issues faced by patients with ostomies, significantly impacting their clinical outcomes and overall quality of life [1,2]. These complications, which include dermatitis, pruritis/xerosis, infections, and ulcerations, often arise due to continuous exposure of the peristomal skin to effluent, mechanical trauma from stoma appliances, and improper stoma care practices [1,2,3,4,5,6,7,8]. Studies indicate that PSCs can affect up to 60% of patients during the first month after surgery, with some reporting even higher rates depending on patient characteristics and care practices [1,4,9].

PSCs often lead to discomfort, reduced self-care confidence, and increased reliance on healthcare services [10]. The financial burden of managing these complications is substantial, with costs driven by repeated consultations, additional medical supplies, and the treatment of secondary infections or injuries [11,12,13,14,15]. Notably, PSCs are known to reduce adherence to stoma care protocols, further exacerbating patient outcomes [12,13,14,15]. Despite the prevalence of PSCs, their prevention remains suboptimal due to a lack of personalized interventions tailored to patient risk profiles [4,7,9]. Current strategies are often generalized, failing to address individual factors such as body mass index (BMI), comorbidities, and ostomy type, which are known to influence the risk of complications [1,3,16]. This gap highlights the urgent need for structured, patient-specific interventions to mitigate PSCs and improve the quality of life for ostomy patients. In this context, risk-stratified protocols, which categorize patients based on their likelihood of developing PSCs, offer a promising solution [4].

The Dermamecum protocol was developed as a structured, risk-stratified educational intervention designed to address the limitations of generalized approaches in PSC prevention [9]. This protocol categorizes patients into three distinct risk groups—green, yellow, and red—based on their likelihood of developing PSCs. The stratification is determined through a comprehensive assessment of pre-operative and post-operative characteristics, including factors such as BMI, comorbidities, stoma type, and prior treatments [9]. Patients on the green path are identified as low-risk and receive basic education focused on sustaining healthy behaviors and routine stoma care. The yellow path targets moderate-risk patients, providing additional education on recognizing early signs of complications and emphasizing self-monitoring. For patients in the red path who are considered high-risk, the protocol incorporates intensive monitoring, frequent follow-ups, and direct access to specialized healthcare support [9].

While the Dermamecum protocol introduces a promising risk-stratified approach to preventing PSCs, the evidence supporting its broader impact remains limited [9]. Interim analyses have provided insights into the protocol’s feasibility and initial outcomes [9]; however, a comprehensive evaluation of its effects by employing longer follow-up periods is lacking. Specifically, questions remain regarding how effectively the protocol mitigates early and late PSCs, enhances patient-reported self-care practices, and improves quality of life over time [9]. Additionally, the ability of the Dermamecum protocol to tailor interventions effectively across risk groups (green, yellow, and red) requires further validation to ensure it meets the specific needs of patients at varying levels of risk. For these reasons, the aim of this study was to evaluate the effects of the Dermamecum protocol, a risk-stratified educational intervention, on preventing PSCs in patients with ostomies over a 90-day follow-up period. The effects were assessed by comparing early (30 days) and late (60 and 90 days) complications, patient-reported self-care practices (maintenance, monitoring, management, and confidence), and quality of life metrics across stratified risk groups (green, yellow, and red paths), tailored to individual patient needs and risk levels.

## 2. Materials and Methods

### 2.1. Design

This study employed a prospective, observational cohort design to evaluate the effects of the Dermamecum protocol, a risk-stratified educational intervention, on preventing PSCs in patients with ostomies. The study included patients from multiple centers over a 90-day follow-up period, with assessments conducted at baseline, 30 days, 60 days, and 90 days. The reporting of this study has been conducted in accordance with the Strengthening the Reporting of Observational Studies in Epidemiology (STROBE) guidelines for cohort studies to ensure methodological rigor and transparency [17].

### 2.2. Setting

The study was conducted within the framework of the Skin Health Academy, a network of Italian stoma care services established in early 2020 [4,9]. This network comprises specialized stoma care nurses and dermatologists who deliver standardized, proactive educational approaches to reduce PSCs [3,10]. The Dermamecum protocol, the primary focus of this study, was developed through three years of collaborative efforts and educational initiatives within the network and became operational in 2023 as routine care in the centers adhering to the Skin Health Academy.

The protocol is implemented in the post-operative phase, typically beginning two weeks after surgery. During this period, specialized nurses tailor interventions to the risk profile of each patient, as determined by pre-operative and post-operative assessments. The program spans up to three months post-surgery, allowing for comprehensive monitoring, education, and support. While future iterations may aim to initiate the protocol earlier in the care continuum, the current timing ensures consistent application across the network.

Patients receive care according to the Dermamecum protocol. It represents a structured, risk-stratified educational approach tailored to each patient’s unique needs, with interventions and follow-ups varying according to their stratified risk group (green, yellow, or red). This approach ensures personalized care and aligns educational efforts with patient-specific risks. The Skin Health Academy also emphasizes pre-operative engagement, where possible, with nurses visiting patients to conduct stoma siting and establish a rapport before surgery [18]. This proactive approach aims to build trust and prepare patients for their post-operative care journey. The Dermamecum protocol leverages these foundational interactions to enhance its impact, focusing on preventing complications and promoting self-care behaviors from the outset.

### 2.3. Risk Stratification-Based Educational Approach (The Dermamecum Protocol)

In the Dermamecum protocol, patients are categorized into three distinct risk paths—green, yellow, and red—based on their likelihood of developing PSCs, which is assessed during their first visit with a specialist nurse approximately two weeks after stoma formation. This stratification is determined through a comprehensive evaluation of demographic, clinical, and post-operative characteristics, including BMI, comorbidities, stoma type, and prior treatments. The Dermamecum protocol ensures that each patient receives tailored education and support that is aligned with their specific risk profile. The approach promotes effective self-care, timely intervention, and improved clinical outcomes by stratifying patients into risk-based paths [1,3,5,6,7,16,19]. It also addresses the limitations of generalized educational programs by targeting interventions to the unique needs of each risk group, thereby reducing the incidence and severity of PSCs.

More precisely, the protocol calculates four specific risk scores corresponding to different types of complications (dermatitis, pruritus/xerosis, infection, and ulceration), using a set of predictive and protective factors drawn from the literature [4,9]. For each complication, the risk is derived from a set of empirically validated predictors and protective factors [4,9]. The risk of dermatitis increases in the presence of psoriasis, chemotherapy, one-piece devices, and an irregular stoma profile, whereas the use of hydrocolloid plaques, low pH detergents, and a low-residue diet are protective. Pruritis/xerosis is associated with overweight/obesity, male sex, and deep convex plaques, while older age and a sedentary lifestyle are protective. Infection risk is elevated in patients with Class 2 obesity, hernias, frequent physical activity, or caregiver dependency and is reduced with one-piece devices. Ulceration is linked to underweight status, non-autonomy, IBD, and radiotherapy, with protective effects from regular abdominal profiles, everted stoma form, and the use of protective film.

Each patient’s risk for the four complications is calculated, and the arithmetic mean of these values defines the overall risk score. Patients are then stratified into three risk paths—green (<25%), yellow (25–75%), and red (>75%)—with each path linked to a tailored educational and monitoring strategy.

#### 2.3.1. Green Path

Patients in this group are considered low-risk, with an overall PSC risk of less than 25%. The objective for these patients is to maintain their current health status through basic self-care education. This includes guidance on proper stoma cleaning, correct appliance fitting, and routine care practices. Educational materials for this group are simple and focus on reinforcing healthy behaviors.

#### 2.3.2. Yellow Path

Patients with a moderate PSC risk of 25–75% are assigned to this path. Education for this group builds upon the green path by introducing strategies for self-monitoring, recognizing early signs of complications, such as dermatitis or infections, and understanding when to seek medical advice. This approach empowers patients to proactively manage their care and address potential complications before they escalate.

#### 2.3.3. Red Path

Patients in this group are at high risk, with a PSC risk exceeding 75%. Interventions for these patients involve stringent monitoring, frequent follow-ups (in person or via phone), and immediate access to healthcare providers. Patients in the red path receive advanced education on recognizing warning signs of severe complications and are given clear instructions for contacting healthcare providers promptly. This intensive approach aims to prevent severe complications through close monitoring and rapid response to symptoms.

### 2.4. Study Procedure

The study procedure was designed to follow the implementation and evaluation of the Dermamecum protocol in routine clinical practice over a 90-day follow-up period. Patients undergoing ostomy surgery at participating Skin Health Academy network centers were screened for eligibility. Baseline data, including demographic (e.g., age, sex, BMI), clinical (e.g., comorbidities, stoma type), and pre-operative characteristics, were collected during the initial post-operative visit (approximately two weeks after stoma formation). Patients were assessed and stratified at this visit into one of the three risk groups (green, yellow, or red) based on their likelihood of developing PSCs. After stratification, patients were enrolled in the Dermamecum protocol. In the green path, patients received basic self-care education focusing on stoma cleaning, appliance fitting, and maintaining healthy behaviors. In the yellow path, patients were educated on self-monitoring techniques to identify early signs of complications, such as dermatitis or infections, and advised on when to seek medical assistance. In the red path, high-risk patients received intensive support, including frequent follow-ups (either in person or remotely) and immediate access to specialized nurses. They were trained to recognize advanced warning signs and given instructions for prompt healthcare engagement.

Patients were followed over a 90-day period, with assessments conducted at baseline (two weeks post-surgery), 30 days, 60 days, and 90 days. During follow-ups, data were collected on the following: (a) the occurrence of early (30 days) and late (60 and 90 days) peristomal complications (e.g., dermatitis, pruritis, infections, or ulcerations); (b) patient-reported self-care outcomes, including maintenance, monitoring, management, and confidence, measured using validated tools; (c) quality of life.

Data collected according to the study procedure are described in Table 1.

Educational materials and support strategies were reinforced, and additional interventions were provided if necessary, particularly for patients in the yellow and red paths. All data were recorded by specialized stoma care nurses using standardized forms. Data included demographic details, clinical assessments, patient-reported outcomes, and observations on PSC prevention. To ensure consistency, nurses participating in the study were trained in the protocol’s implementation and data collection procedures.

#### Ethical Considerations

Study-specific patient consent was waived as the data were collected as part of routine clinical care and did not involve any additional interventions beyond standard practice. All data were fully anonymized to ensure patient confidentiality, making individual identification impossible. Furthermore, the routine patient admission informed consent explicitly included a provision allowing for the use of anonymized clinical data for scientific research purposes, including prospective follow-up assessments. As a result, no additional informed consent was required, and all data handling complied with ethical and legal standards.

### 2.5. Eligibility Criteria

Patients were eligible for inclusion in the study if they were adults aged 18 years or older, had undergone ostomy surgery resulting in the creation of a colostomy, ileostomy, or urostomy within a maximum of two weeks from the risk stratification procedure, and were receiving care from specialized stoma care nurses affiliated with the Skin Health Academy network. Eligible patients had no pre-existing PSCs at the time of enrollment for this study.

Patients were excluded from the study if they presented with pre-existing PSCs prior to the initiation of the Dermamecum protocol, had cognitive impairments or other conditions that could interfere with their ability to comprehend or participate in the educational program, or were unwilling or unable to complete follow-up assessments over the 90-day study period.

### 2.6. Outcomes

The primary outcome of this study was the occurrence of early complications, defined as PSCs observed within the first 30 days following ostomy surgery [9]. Secondary outcomes included late complications, assessed at 60 and 90 days post-surgery, as well as patient satisfaction with the Dermamecum protocol, which was evaluated at 60 days.

Self-care behaviors were also assessed at 30 and 60 days, including self-care maintenance, monitoring, management, and confidence [20]. Additionally, quality of life metrics were evaluated using the Short Form-36 (SF-36) tool [21]. Physical component and mental component scores were recorded at baseline, 30 days, and 90 days.

### 2.7. Measurements

Baseline data included age, sex, BMI, and comorbidities such as diabetes, inflammatory bowel disease (IBD), cardiovascular disorders, and dermatitis-related conditions (e.g., psoriasis, atopic dermatitis, and allergic dermatitis). Previous treatments, including neoadjuvant chemotherapy or radiotherapy, and the type of ostomy created (colostomy, ileostomy, or urostomy) were also documented.

The stoma’s physical characteristics were assessed, including the abdominal profile (regular, extroflexed, or introflexed) and the stoma’s physical form (normal, extroflexed, flat, or introflexed). The implementation of a pre-planned stoma siting was recorded. Post-surgical complications such as hernias, prolapses, and incisional hernias were documented during follow-ups.

Self-care practices were assessed at 30 and 60 days using a validated tool, which is the Ostomy Self-Care Index (OSCI) [20]. The OSCI has demonstrated strong psychometric properties, including a high internal consistency (Cronbach’s alpha > 0.90 for all domains) and construct validity. The tool is sensitive to differences in self-care behaviors and their impact on clinical outcomes, such as PSCs and quality of life. This tool is grounded in the theory of self-care for chronic illness and comprises four distinct domains [22,23]: self-care maintenance, self-care monitoring, self-care management, and self-care confidence.

Each domain evaluates specific self-care behaviors critical to managing and preventing PSCs. The OSCI uses a 5-point Likert scale for responses, ranging from “never” to “always”, with higher scores indicating better self-care performance. Self-care maintenance evaluates patients’ adherence to daily stoma care routines designed to maintain physical and emotional stability. These behaviors include proper cleaning of the stoma and peristomal skin, the appropriate use and maintenance of stoma appliances, and adherence to dietary recommendations. Self-care monitoring measures the extent to which patients observe and recognize changes in the stoma or peristomal skin. This includes monitoring for leaks, appliance condition, changes in output (e.g., feces or urine), and skin conditions around the stoma, as well as observing the effects of diet on stoma output. Self-care management assesses how patients respond to identified issues or changes, such as modifying dietary or fluid intake, adjusting stoma care routines, or seeking timely guidance from healthcare providers. This domain reflects problem-solving behaviors and the ability to address complications effectively. Self-care confidence captures patients’ belief in their ability to manage and maintain their stoma and peristomal skin effectively, persist in self-care behaviors even under challenging circumstances, and assess the success of interventions to resolve complications.

Patient quality of life was assessed using the SF-36, a widely validated tool for measuring health-related quality of life (HRQoL) across multiple dimensions [21]. The SF-36 evaluates eight health domains: physical functioning, role limitations due to physical health problems, bodily pain, general health perceptions, vitality, social functioning, role limitations due to emotional problems, and mental health. Each domain score is transformed into a 0–100 scale, with higher scores indicating a better health status. For this study, the physical and mental health components of the SF-36 were specifically analyzed at baseline, 30 days, and 90 days post-surgery to evaluate the impact of the Dermamecum protocol on overall health and well-being. The Italian version of the SF-36, which has demonstrated strong psychometric properties and cultural adaptability in previous studies, was used to ensure reliability and validity in this population [24].

Early complications (within 30 days) and late complications (at 60 and 90 days) were recorded based on clinical assessments performed by specialized nurses [9]. Patient satisfaction with the Dermamecum protocol was assessed at 60 days by using a single item employing a five-point Likert scale [25]. All measurements were performed by trained stoma care nurses using standardized forms. Nurses received protocol-specific training to ensure consistent and accurate data collection across multiple centers.

### 2.8. Sample Size

Sample size calculations for this study were performed using G*Power Version 3.1.9.6 [26]. As reported in a recent systematic review [1], the protocol estimated the required sample size based on an expected proportion of PSCs of 35% and aimed for a reduction of 9.2% to achieve a PSC rate equal to the first quartile of the PSCs’ distribution (25.8%) [1]. Using a binomial test for a single proportion, the sample size calculation was conducted with the following parameters: a two-tailed test, an effect size (*g*) of 9.2%, a significance level (*α*) of 0.05, a power (1 − *β*) of 0.90, and a constant proportion of 35%. The results of the calculation indicated that the required total sample size was 300 participants. Following the interim analysis [9], it was observed that the reduction in PSCs was anticipated to be greater than originally estimated, with a roughly 28% reduction (from 35% to 7%).

However, instead of halting data collection, it was deemed important to continue the study while updating the sample size calculation [27]. This approach ensured adequate power to perform a Poisson regression analysis to evaluate the effects of each path on PSC rates. The updated sample size calculation utilized G*Power for Poisson regression (z-test), considering the following parameters: a two-tailed test, Exp(β₁) = 1.87 (indicating an 87% increase in risk for high-risk patients compared to the green path group), a base rate of Exp(β₀) = 0.075 (calculated from preliminary interim data), a significance level (α) of 0.05, a power (1 − *β*) of 0.90, and a mean exposure of 1. The predictors were assumed to follow a normal distribution with a mean (*μ*) of 0 and a standard deviation (*σ*) of 1, with *R*^2^ from other predictors set to 0 [28]. Based on these parameters, the updated sample size calculation indicated that a total of 305 participants were required.

### 2.9. Data Analysis

Data were summarized based on their nature. Frequencies and percentages were used to describe categorical variables. Quantitative variables were reported as the mean ± standard deviation (SD) if normally distributed and as the median with interquartile range (IQR) if non-normally distributed.

Comparative analyses were performed to assess differences in primary outcomes (PSCs) and secondary outcomes (self-care, HRQoL, and satisfaction only at T2) across groups at each available time point. For inferential comparisons, *p*-values were corrected for multiple testing using the Benjamini–Hochberg (BH) procedure to control the false discovery rate. Additionally, trend analyses for continuous and categorical outcomes across time points were conducted using appropriate statistical tests.

A Poisson regression analysis was performed to evaluate the temporal trends in PSCs. The outcome variable was the total number of PSCs at each time point (T0, T1, T2, and T3). Predictors included time (treated as a categorical variable), path assignment, and other patient-specific factors (i.e., sex, age, and BMI). Patient-specific factors were selected based on their clinical relevance and prior evidence in the literature [9]. The results were presented as incidence rate ratios (IRRs), regression coefficients, standard errors (SE), z-values, and *p*-values. Model fit was assessed using deviance and Pearson χ^2^ statistics, with corresponding *p*-values to ensure goodness-of-fit. Overdispersion was assessed by examining the deviance-to-degrees-of-freedom ratio. The ratio remained close to 1 in both the initial and stepwise models, suggesting that overdispersion was not a concern and that the Poisson model assumptions were adequately met. All analyses were performed using R 4.2.2, supported by appropriate libraries.

## 3. Results

### 3.1. Sample Characteristics

The study included a total of 305 participants, stratified into three risk-based paths as per the Dermamecum protocol: the green path (n = 43; 14.1%), the yellow path (n = 204; 66.9%), and the red path (n = 58; 19.0%). Table 2 summarizes the baseline characteristics of the sample and provides a comparison across the three groups.

The overall sample had a nearly balanced sex distribution, with 56.7% male and 43.3% female participants overall. However, significant sex-based differences were observed between the groups, with the green path including a higher proportion of females (79.1%) compared to males, while the yellow and red paths had a higher proportion of males (64.2% and 56.9%, respectively; *p* < 0.001).

The mean age of participants was 65.75 years (SD = 14.11), with no statistically significant difference across the groups (*p* = 0.251). Regarding BMI, most participants were in the normal weight category (51.8%), though a higher prevalence of obesity (19.0%) and underweight status (17.2%) was observed in the red path (*p* < 0.001).

Comorbidities such as diabetes (13.4%), IBD (6.9%), and cardiovascular disorders (38.0%) were reported, with the yellow path having the highest prevalence of diabetes (16.7%; *p* = 0.003), and the red path showing a notably higher proportion of IBD cases (19.0%). A total of 54.4% of participants reported at least one comorbidity, with no significant differences across groups (*p* = 0.898).

Among previous treatments, 22.0% received neoadjuvant chemotherapy, with the yellow path showing the highest proportion (27.5%; *p* = 0.004). Regarding stoma type, colostomies (40.3%) and ileostomies (39.7%) were the most common, with the red path including the highest proportion of ileostomies (56.9%; *p* = 0.009).

Pre-operative stoma siting was scheduled for 79.5% of participants, with the highest adherence in the green path (90.7%; *p* = 0.036). Notably, participants in the green path were more likely to have a regular abdominal profile (90.7%), compared to 66.2% in the yellow path and 25.9% in the red path (*p* < 0.001).

Among patients, 37.4% were not working, with the red path having the highest proportion of non-working individuals (46.6%), followed by the green path (37.2%) and the yellow path (34.8%). Semi-sedentary jobs were the most common among those employed, with 30.2% overall and the yellow path having the highest proportion (32.8%). Sedentary and non-sedentary jobs were less common, but participants in the green path were more likely to have sedentary jobs (18.6%; *p* = 0.009) than the other paths.

Over half of the participants (55.7%) reported never engaging in physical activity, with the red path showing the highest proportion (65.5%), followed by the green path (58.1%) and yellow path (52.5%). Regular sports activity was rare, with only 4.6% of participants engaging in sports “always”, and no significant differences were observed across paths (*p* = 0.194).

#### Baseline Assessments

At the baseline (two weeks after stoma formation), the scheduled stoma site was implemented in 72.5% of participants, with the highest proportion observed in the green path (83.7%), followed by the yellow path (73.5%), and the lowest in the red path (60.3%; *p* = 0.029).

Dietary recommendations varied across the sample. A low-residue diet was prescribed for 35.4% of participants, with the green path having the highest adherence (51.2%) and the red path the lowest (20.7%; *p* = 0.027). Diets tailored for ileostomy were most common in the red path (43.1%), reflecting this group’s higher proportion of ileostomy procedures.

Participants in the green path predominantly had a regular abdominal profile (90.7%), compared to 66.2% in the yellow path and only 25.9% in the red path (*p* < 0.001). Extroflexed and introflexed profiles were more common in the red path (43.1% and 31.0%, respectively). The overall rates of complications such as incisional hernias (2.3%), prolapse (1.6%), and hernias (0.3%) were low, with no significant differences observed across the groups (*p* = 0.424).

Participants were equally divided between single-piece (48.9%) and two-piece devices (50.2%), with no significant differences across the paths (*p* = 0.629). Most participants used traditional convex baseplates (60.0%), while deep convex baseplates were rare, being most common in the green path (9.3%; *p* = 0.120). Neutral pH soap was the most commonly used hygiene product (58.3%), with similar usage across paths (*p* = 0.640). Non-woven gauze was more frequently used in the green path (62.8%), while wet wipes were less common overall (17.9%).

### 3.2. Early Onset Complications (30 Days)

At 30 days post-surgery, the overall rate of early-onset PSCs across the cohort was 8.5% (26 out of 305 patients).

**Figure 1:**
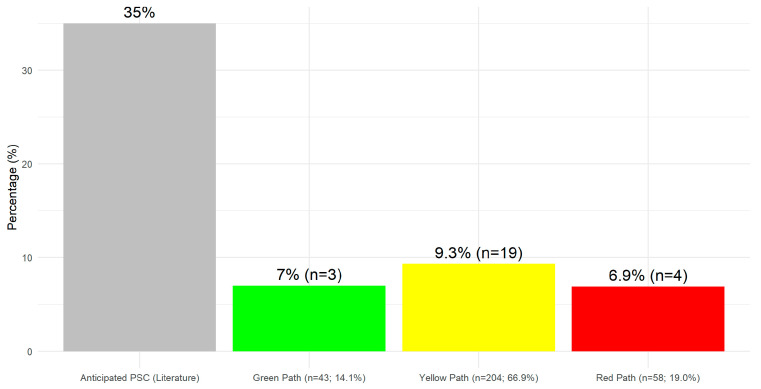
PSCs in each path of the Dermamecum protocol (30 days).

Figure 1 provides the comparison of early-onset PSCs across different risk paths (and the anticipated complication rate from the literature) [1]. Among the 26 patients who experienced PSCs, 7.0% (n = 3) were from the green path, 9.3% (n = 19) from the yellow path, and 6.9% (n = 4) from the red path.

These observed rates are notably lower than the anticipated PSC rate of 35% reported in the literature, highlighting the overall effectiveness of the intervention. No significant difference in PSC rates was detected (*p* = 0.545).

### 3.3. Late-Onset Complications

#### 3.3.1. PSCs at 60 Days

**Figure 2:**
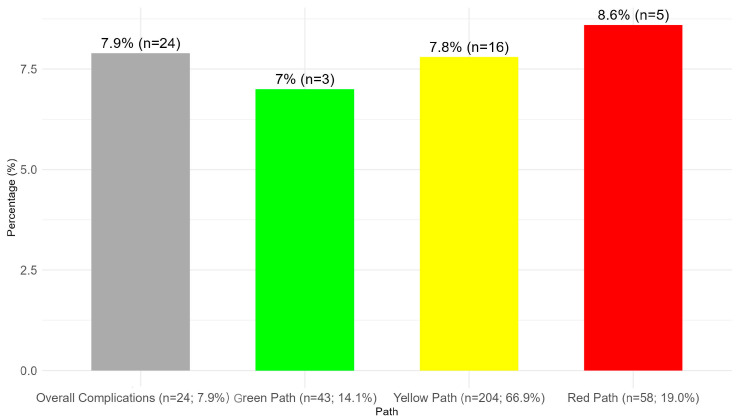
PSCs in each path of the Dermamecum protocol (60 days).

At 60 days post-surgery, the overall late-onset PSC rate was 7.9% (24 out of 305 patients). When stratified by path, the rates were 7.0% (3 out of 43) in the green path, 7.8% (16 out of 204) in the yellow path, and 8.6% (5 out of 58) in the red path, as illustrated in Figure 2. No significant difference in PSC rates was detected (*p* = 0.804).

#### 3.3.2. PSCs at 90 Days

**Figure 3:**
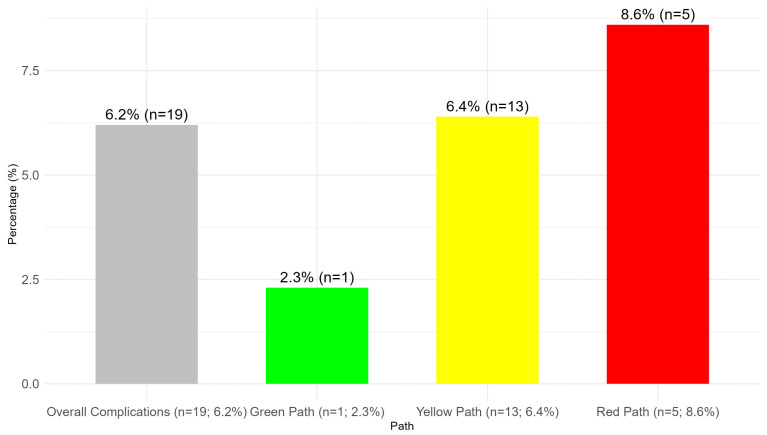
PSCs in each path of the Dermamecum (90 days).

At 90 days post-surgery, the overall incidence of late-onset PSCs was 6.2% (19 out of 305 patients). The distribution of complications across the different paths showed that green path patients experienced the lowest complication rate (2.3%, n = 1), followed by yellow path patients (6.4%, n = 13) and red path patients (8.6%, n = 5), as illustrated in Figure 3 (*p* = 0.466).

#### 3.3.3. Trends in PSCs over Time

**Figure 4:**
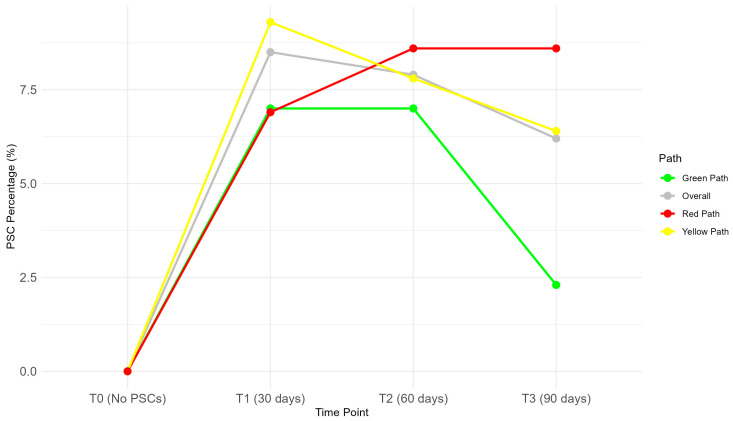
Trends in PSCs over time.

The progression of PSCs over time is displayed in Figure 4. The overall rate of PSCs decreased from T1 (30 days) to T2 (60 days) and then further declined at T3 (90 days).

The green path exhibited the lowest PSC rates over time for path-specific trends, with a significant trend (*p* = 0.004). The yellow path demonstrated a significant decrease in PSC rates over time (*p* = 0.017) as well, while the red path displayed a weaker but statistically significant trend indicating a stable proportion of PSCs in T2 and T3, which were slightly increased compared to T1 (*p* = 0.049). The overall trend, combining all paths, was highly significant (*p* = 0.002), suggesting an overall increase in PSC rates during the first 60 days post-surgery, followed by stabilization.

### 3.4. Self-Care

#### 3.4.1. Self-Care at 30 Days

The median self-care maintenance score was 94.44 (IQR: 86.11–100) across the cohort, with consistent results observed across the green, yellow, and red paths (*p* = 0.730). The median score for self-care monitoring was 87.5 (IQR: 75.00–96.88). The green path reported a median of 85.93 (IQR: 78.13–96.80), the yellow path 87.5 (IQR: 75.00–96.87), and the red path 89.06 (IQR: 75.00–96.88), with no significant differences between the paths (*p* = 0.865).

For self-care management, the overall median score was 90.63 (IQR: 78.13–100). The green path showed a median of 89.06 (IQR: 75.00–96.88), the yellow path 92.19 (IQR: 81.25–100), and the red path 93.75 (IQR: 78.13–100). No significant differences were found between the paths (*p* = 0.446). The self-care confidence domain recorded a median score of 80.0 (IQR: 65.00–95.00) overall, with the green path reporting 75.00 (IQR: 57.50–92.50), the yellow path 80.00 (IQR: 67.50–95.00), and the red path 80.00 (IQR: 65.00–97.50). Differences between paths in this domain were also not statistically significant (*p* = 0.365).

#### 3.4.2. Self-Care at 60 Days

The median self-care maintenance score for the overall cohort was 97.22 (IQR: 88.89–100). The green path reported a median of 98.6 (IQR: 88.19–100), the yellow path with 97.22 (IQR: 88.89–100), and the red path with 94.44 (IQR: 83.33–100). No statistically significant differences were observed between the paths (*p* = 0.675).

The median self-care monitoring score for the overall cohort was 90.63 (IQR: 78.13–100). The yellow path recorded a median of 92.9 (IQR: 81.3–100.0), the red path a median of 93.75 (IQR: 78.13–100), and the green path a median of 89.06 (IQR: 75.0–96.88). The differences across paths were not statistically significant (*p* = 0.694).

For self-care management, the overall median score was 87.5 (IQR: 75.0–96.87). The red path had a median of 89.06 (IQR: 75.0–96.88), while the green path reported a median of 85.94 (IQR: 78.13–96.9), and the yellow path aligned with the overall median. There were no significant differences between paths (*p* = 0.899).

In the self-care confidence domain, the overall median was 87.5 (IQR: 72.5–97.5). The red path reported a median of 92.5 (IQR: 75.0–100.0), while the yellow path matched the overall median, and the green path had a median of 78.75 (IQR: 65.0–90.63). Despite this variation, the differences were not statistically significant (*p* = 0.146).

#### 3.4.3. Self-Care: Trends

The trends in self-care domains between 30 days (T1) and 60 days (T2) post-surgery reveal descriptive changes overall and across specific paths, though no statistically significant differences were observed across time points. Self-care maintenance showed a slight overall improvement (94.44 to 97.22, *p* = 0.675), with the largest increase observed in the green path (94.44 to 98.60). Self-care monitoring improved modestly overall (87.50 to 90.63, *p* = 0.694), with the yellow path showing the greatest increase (87.50 to 92.90). Self-care management scores declined slightly overall (90.63 to 87.50, *p* = 0.899), with similar reductions across paths. Self-care confidence improved overall (80.00 to 87.50, *p* = 0.146), with the red path showing the most notable increase (80.00 to 92.50). These changes, however, were not statistically significant.

### 3.5. HRQoL

#### 3.5.1. HRQoL at Baseline

At baseline, the physical component of the SF-36 had a median score of 35.2 (IQR: 31.2–41.14) overall, with slight variations across paths: 34.49 (IQR: 31.03–40.2) for the green path, 35.86 (IQR: 31.4–41.68) for the yellow path, and 34.12 (IQR: 31.07–38.29) for the red path (*p* = 0.224). The mental component had a median score of 38.52 (IQR: 35.77–41.84) overall, with path-specific scores of 37.77 (IQR: 35.82–42.2) for the green path, 38.71 (IQR: 35.77–41.89) for the yellow path, and 38.72 (IQR: 35.77–41.66) for the red path (*p* = 0.988).

#### 3.5.2. HRQoL at 30 Days (T1)

At 30 days post-surgery, the physical component score slightly declined overall to 34.38 (IQR: 30.39–39.3), with scores of 35.98 (IQR: 32.72–38.5) for the green path, 34.38 (IQR: 30.27–39.60) for the yellow path, and 33.97 (IQR: 30.9–39.57) for the red path (*p* = 0.711). The mental component score remained stable overall at 38.19 (IQR: 35.58–41.06), with scores of 37.53 (IQR: 35.28–39.7) for the green path, 38.23 (IQR: 35.77–41.93) for the yellow path, and 37.50 (IQR: 34.78–40.11) for the red path (*p* = 0.238).

#### 3.5.3. HRQoL at 90 Days (T2)

At 90 days post-surgery, the physical component improved slightly overall to 35.7 (IQR: 31.1–39.60), with scores of 34.39 (IQR: 30.41–39.9) for the green path, 36.62 (IQR: 31.70–39.60) for the yellow path, and 34.29 (IQR: 29.83–39.60) for the red path (*p* = 0.625). The mental component score slightly decreased overall to 38.00 (IQR: 35.77–40.46), with scores of 37.86 (IQR: 34.93–40.3) for the green path, 38.14 (IQR: 35.77–41.13) for the yellow path, and 38.00 (IQR: 36.98–39.22) for the red path (*p* = 0.891).

#### 3.5.4. Trends in HRQoL

From baseline to 90 days post-surgery, descriptive HRQoL trends were observed across the physical and mental components of the SF-36, but no statistically significant changes were detected over time (all *p*-values > 0.05). The physical component remained relatively stable, with minor fluctuations across paths and *p*-values of 0.812, 0.756, 0.697, and 0.662 for the overall cohort and the green, yellow, and red paths, respectively. Similarly, the mental component showed minimal variation, with consistent median scores and overlapping interquartile ranges across time points, with *p*-values of 0.901, 0.834, 0.882, and 0.875 for the overall cohort and the green, yellow, and red paths, respectively.

### 3.6. Satisfaction (Assessed at T2)

Patient satisfaction with the Dermamecum protocol was assessed at 60 days post-surgery (T2). The overall median satisfaction score was 5.00 (IQR: 4.00–5.00). Across the different paths, the green path, yellow path, and red path exhibited median scores of 5.00 (IQR: 4.00–5.00), 5.00 (IQR: 4.00–5.00), and 4.00 (IQR: 3.00–5.00), respectively. No statistically significant differences in satisfaction scores were observed among the paths (*p* = 0.203).

### 3.7. Poisson Regression for Temporal Trends in PSCs

The deviance (38.2, *p* = 0.642) and Pearson χ^2^ (40.1, *p* = 0.718) tests indicated a good model fit, suggesting that the Poisson model assumptions were adequately met and overdispersion was not a concern.

As shown in Table 3, at 30 days (T1), the first observed PSC incidence was 1.243 times the reference rate (*p* = 0.011), marking the onset of complications. By 60 days (T2), the complication rate continued to rise, showing a 36.6% increase compared to T1 (IRR = 1.366, *p* < 0.001). At 90 days (T3), the rate remained elevated but slightly lower than at T2, with a 22.3% higher risk compared to T1 (IRR = 1.223, *p* = 0.014).

Path assignment was a significant predictor of PSC rates. Patients in the green path had a significantly lower risk of PSCs compared to the reference group (IRR = 0.636, *p* < 0.001). However, no significant difference was observed for the yellow path (*p* = 0.253).

Among patient-specific factors, age and BMI were significant predictors of PSC occurrence. Each additional year of age was associated with a 2.1% increase in PSC risk (IRR = 1.021 per year, *p* < 0.001). Overweight patients had a 17.0% higher risk (IRR = 1.170, *p* = 0.029), while obese patients exhibited a 28.5% higher risk (IRR = 1.285, *p* = 0.003) compared to those with a normal BMI. No significant association was found between sex and PSC risk (*p* = 0.183).

## 4. Discussion

This study evaluated the effects of the Dermamecum protocol, a risk-stratified educational intervention, on preventing PSCs and improving self-care, HRQoL, and patient satisfaction over a 90-day follow-up period. The primary outcome, PSC rates, showed that overall incidence remained low across all time points, with early complications observed in 8.5% of participants at 30 days and late complications at 7.9% and 6.2% at 60 and 90 days, respectively. No statistically significant differences in PSC rates were observed between the risk-based paths. These results, showing a longitudinal description from baseline to 90 days, represent a novelty in the available description of PSC rates in the literature as studies aimed at their reporting are generally cross-sectional [15,29,30,31,32,33]. In line with recent calls to improve person-centered care for individuals with an ostomy [15,16,34], this study offers actionable evidence that may contribute to enhancing their quality of life, as also recognized by the peer reviewers.

For secondary outcomes, self-care scores improved in maintenance, monitoring, and confidence domains over time, though no statistically significant differences were detected across paths or time points. HRQoL remained stable, with minor physical and mental component score fluctuations, but without significant variations over time or between groups. Patient satisfaction, assessed at 60 days, was high across all paths, reflecting the perceived acceptability and usability of the Dermamecum protocol. The findings from the Poisson regression analysis highlight a significant temporal progression in PSC rates over time. The risk of PSCs increased notably at 30, 60, and 90 days post-surgery, indicating a pattern of progressive complication development, as anticipated.

These results suggest that while PSCs may initially emerge within the first month, their incidence rises before stabilizing in later stages. This trend underscores the importance of ongoing monitoring and tailored interventions beyond the immediate post-operative period to mitigate long-term complications. Among patient-specific factors, age, BMI, and risk path (with the green path showing a lower risk) were significant predictors of PSCs, underscoring the importance of these variables in tailoring preventive interventions. These findings emphasize the utility of a structured, risk-stratified approach in managing and preventing PSCs while maintaining high levels of patient satisfaction and stable HRQoL outcomes [15,16,34].

The observed PSC rates in this study are lower than previous findings, where complications ranged from 35% to 73.4% [1,34]. Reducing early PSCs suggests that structured, risk-based educational interventions could effectively mitigate these complications. This aligns with the international consensus emphasizing the importance of preventive measures in ostomy care [35].

Regarding self-care outcomes, the observed stability over time was anticipated, and it is coherent with the behavioral nature of self-care practices [8]. Self-care behaviors, such as stoma maintenance, monitoring for complications, and management strategies, are often rooted in routine and habit formation. Once patients acquire the necessary skills and confidence through structured education, such as the Dermamecum protocol, these practices tend to persist over time. This stability is critical in ensuring long-term adherence to effective self-care strategies, as it reduces the likelihood of complications and promotes autonomy in managing ostomy-related challenges [36,37,38,39]. Additionally, the consistency observed in self-care outcomes underscores the importance of early, comprehensive educational interventions to reinforce these behaviors during the initial post-surgical period, when patients are most receptive to guidance and support [40].

The stability of HRQoL observed in this study is encouraging, as PSCs are known to adversely affect HRQoL, particularly in areas such as physical discomfort, emotional well-being, and social functioning [41,42]. The Dermamecum protocol likely contributed to maintaining HRQoL by addressing several critical aspects of care. Firstly, the risk-stratified approach ensures that patients at a higher risk of complications (e.g., those in the red path) receive more intensive monitoring, education, and support. This proactive care may help to prevent severe complications, reduce physical discomfort, and enhance patients’ ability to manage their condition effectively [43,44,45]. Secondly, the protocol’s emphasis on tailored education plays a vital role in empowering patients to feel confident and capable in their self-care practices [39]. The protocol fosters a sense of control and autonomy, which are crucial for emotional well-being, by providing practical, individualized guidance on stoma care, recognizing early signs of complications, and understanding when to seek professional support. Moreover, the Dermamecum protocol’s structured follow-ups and accessibility to specialized care may alleviate patients’ anxiety about managing their ostomy, particularly for those in higher-risk categories [46]. Knowing they have direct access to specialized stoma care nurses reduces feelings of isolation and builds a support network that extends beyond hospital discharge. In addition, the focus on patient satisfaction as part of the intervention likely contributed to better psychological outcomes [47].

The results of this study emphasize the importance of integrating risk-stratified approaches, such as the Dermamecum protocol, into routine clinical practice to prevent PSCs, enhance self-care, and maintain HRQoL. Tailored education and proactive monitoring are essential, particularly for high-risk patients, to promote autonomy and reduce complications [48]. Addressing modifiable risk factors, such as BMI, and providing accessible follow-ups through specialized stoma care nurses can ensure timely interventions and reduce patient anxiety. High patient satisfaction highlights the value of patient-centered care that addresses both clinical and psychological needs. Integrating digital health tools for self-monitoring and expanding educational strategies to further improve outcomes can enhance patient engagement and streamline care delivery. These strategies underscore the critical role of personalized, proactive, and resource-efficient interventions in optimizing outcomes for patients with ostomies.

This study has several limitations that should be considered when interpreting the findings. First, the results must be understood within the context of the cohort design, which lacks a control group for direct comparison. Without randomization, the observed effects of the Dermamecum protocol cannot be definitively attributed to the intervention, as other unmeasured factors may have influenced the outcomes [49]. Second, the absence of a control group limits the ability to account for natural variations in PSC rates or outcomes related to standard care practices [50]. While the prospective cohort design offers valuable real-world insights, a comparison with standard care would have strengthened the conclusions. Future studies employing randomized controlled trial designs are recommended to validate the efficacy of the Dermamecum protocol and establish causal relationships. Third, while the risk-stratified approach allowed for tailored interventions, the relatively small sample size within specific paths, particularly the green path, may have reduced the power to detect subtle differences in outcomes [51]. Additionally, the study was conducted within a specific network of stoma care services, which may limit the generalizability of the findings to other healthcare settings or populations [52]. Fourth, reliance on patient-reported outcomes for self-care, HRQoL, and satisfaction may introduce potential biases, such as recall or social desirability bias [53]. Moreover, the observed PSC rate at 30 days (8.5%) was markedly lower than commonly reported in the literature (ranging from 35% to 73%), which, while promising, raises the possibility of selection, measurement, or implementation-related biases [54]. These include heightened nurse expertise in the participating centers, proactive patient engagement, and potential underreporting of mild complications. These factors should be carefully considered when interpreting the apparent effectiveness of the protocol. Lastly, because this study was conducted within the Italian Skin Health Academy network, which is a structured system of highly specialized stoma care services, the findings may reflect specific contextual factors such as nurse expertise, patient education practices, and healthcare delivery models unique to this setting. As such, cultural, organizational, or systemic differences in other countries or healthcare systems may affect the applicability and generalizability of the results. Future research should explore the implementation and outcomes of the Dermamecum protocol in diverse clinical, educational, and cultural contexts to evaluate its broader external validity [55,56]. Despite these limitations, the study provides valuable insights into the potential benefits of risk-stratified educational interventions, which warrant further evaluation through randomized controlled trials to confirm these findings and establish causal relationships.

## 5. Conclusions

This study highlights the potential of the Dermamecum protocol, a risk-stratified educational intervention, in preventing PSCs and improving patient outcomes over a 90-day follow-up period. The protocol demonstrated low PSC rates, stable HRQoL, and high patient satisfaction, tailoring care to individual risk profiles. Future randomized controlled trials are necessary to confirm these results and explore the protocol’s long-term effectiveness. Overall, the Dermamecum protocol underscores the importance of personalized, proactive, and patient-centered approaches in ostomy care, offering a valuable model for improving outcomes in this population.

## Figures and Tables

**Table 1:** Data collected at each time point.

Time Point	Clinical Assessment	PSC ^¶^	Self-Care	HRQoL	Satisfaction
Baseline (T0)	Yes	Yes *	No	Yes	No
T1 (30 days)	Yes	Yes	Yes	Yes	No
T2 (60 days)	Yes	Yes	Yes	Yes	Yes
T3 (90 days)	Yes	Yes	No	Yes	No

* The absence of PSC is an inclusion criterion. **^¶^** All patients reported to have zero PSC at T0.

**Table 2:** Characteristics of the sample, path groups, and comparisons.

		Overall (n = 305)		Green Path (n = 43; 14.1% of the Total)		Yellow Path (n = 204; 66.9% of the Total)		Red Path (n = 58; 19.0% of the Total)		*p* ^¥^
		N	%	N	%	N	%	N	%	
**Pre-operative information**										
Sex										
	Male	173	56.7	9	20.9	131	64.2	33	56.9	<0.001
	Female	132	43.3	34	79.1	73	35.8	25	43.1	
Age										
	Years (mean; SD)	65.75	14.11	68.91	13.68	65.22	14.20	65.29	14.06	0.251
BMI										
	Underweight	33	10.8	2	4.7	21	10.3	10	17.2	<0.001
	Normal	158	51.8	35	81.4	107	52.5	16	27.6	
	Overweight	83	27.2	5	11.6	57	27.9	21	36.2	
	Obese	31	10.2	1	2.3	19	9.3	11	19.0	
Comorbidity										
	Diabetes	41	13.4	3	7.0	34	16.7	4	6.9	0.003
	IBD	21	6.9	2	4.7	8	3.9	11	19.0	
	Hematologic disorders	25	8.2	2	4.7	21	10.3	2	3.4	
	Cardiovascular disorders	116	38.0	15	34.9	83	40.7	18	31.0	
	Psoriasis	1	0.3	0	0	0	0	1	1.7	
	Atopic dermatitis	8	2.6	2	4.7	4	2.0	2	3.4	
	Allergic dermatitis	22	7.2	2	4.7	15	7.4	5	8.6	
	Any	166	54.4	22	51.2	112	54.9	32	55.2	0.898
Previous treatment										
	Neoadjuvant Chemotherapy	67	22.0	6	14	56	27.5	5	8.6	0.004
	Radiotherapy	44	14.4	3	7.0	35	17.2	6	10.3	0.139
Any previous surgeries										
	Yes	88	28.9	13	30.2	57	27.9	18	31.6	0.849
Type of stoma that was planned										
	Colostomy	123	40.3	20	46.5	84	41.2	19	32.8	0.009
	Ileostomy	121	39.7	10	23.3	78	38.2	33	56.9	
	Urostomy	61	20.0	13	30.2	42	20.6	6	10.3	
A stoma siting was scheduled										
	Yes	240	79.5	39	90.7	162	79.8	39	69.6	0.036
Job types										
	Sedentary	20	6.6	8	18.6	8	3.9	4	6.9	0.009
	Semi-sedentary	92	30.2	12	27.9	67	32.8	13	22.4	
	Non-sedentary	79	25.9	7	16.3	58	28.4	14	24.1	
	Not working	114	37.4	16	37.2	71	34.8	27	46.6	
Sport activity										
	Never	170	55.7	25	58.1	107	52.5	38	65.5	0.194
	Rarely	83	27.2	11	25.6	57	27.9	15	25.9	
	Often	38	12.5	7	16.3	29	14.2	2	3.4	
	Always	14	4.6	0	0	11	5.4	3	5.2	
**Post-operative (15 days)–** **Dermamecum baseline**										
Scheduled stoma site implemented										
	Yes	221	72.5	36	83.7	150	73.5	35	60.3	0.029
Diet										
	Low residue	108	35.4	22	51.2	74	36.3	12	20.7	0.027
	For ileostomy	102	33.4	10	23.3	67	32.8	25	43.1	
	No special diet	57	18.7	7	16.3	41	20.1	9	15.5	
	Other	38	12.5	4	9.3	22	10.8	12	20.7	
Abdomen profile										
	Regular	189	62.0	39	90.7	135	66.2	15	25.9	<0.001
	Extroflexed	68	22.3	4	9.3	39	19.1	25	43.1	
	Introflexed	48	15.7	0	0	30	14.7	18	31.0	
Physical form of the stoma post-surgery										
	Extroflexed	95	31.1	10	23.3	69	33.7	16	27.6	0.478
	Normal	81	26.6	14	32.6	48	23.4	19	32.8	
	Low	51	16.7	6	14.0	39	19.0	6	10.3	
	Flat	36	11.8	5	11.6	23	11.2	8	13.8	
	Introflexed	39	12.8	8	18.6	24	11.7	7	12.1	
	Missing data	3	1.0	0	0	1	0.5	2	3.4	
Post-surgical herniation and prolapse complications										
	Incisional hernia	7	2.3	2	4.7	3	1.5	2	3.4	0.424
	Prolapse	5	1.6	0	0	3	1.5	2	3.4	
	Hernia	1	0.3	0	0	1	0.5	0	0	
Device										
	Single-piece device	149	48.9	24	55.8	97	47.5	28	48.3	0.629
	Two-piece device	153	50.2	19	44.2	106	52.0	28	48.3	
	Missing data	3	1.0	0	0	1	0.5	2	3.4	
Device baseplate										
	Flat	109	35.7	15	34.9	71	34.8	23	41.1	0.120
	Traditional convex	183	60.0	24	55.8	126	61.8	33	58.9	
	Deep convex	10	3.3	4	9.3	6	2.9	0	0	
	Missing data	3	1.0	0	0	1	0.5	2	3.4	
Baseplate material										
	Hydrocolloid	230	75.4	28	65.1	155	76.0	47	81.0	0.093
	Hydrocolloid plus nonwoven textile	72	23.6	15	34.9	48	23.5	9	15.5	
	Missing data	3	1.0	0	0	1	0.5	2	3.4	
Caregiver support										
	Full	91	29.8	13	30.2	59	28.9	19	32.8	0.719
	Partial	82	26.9	8	18.6	59	28.9	15	25.9	
	Supervisory	42	13.8	6	14.0	27	13.2	9	15.5	
	None	87	28.5	16	37.2	58	28.4	13	22.4	
	Missing data	3	1.0	0	0	1	0.5	2	3.4	
Ostomy care products										
	Pasta with alcohol	38	12.6	7	16.3	25	12.3	6	10.7	0.810
	Alcohol-free paste	107	35.4	16	37.2	66	32.5	25	44.6	
	Protective film	168	55.6	23	53.5	111	54.7	34	60.7	
	Remover utilization	261	86.4	36	83.7	176	86.7	49	87.5	
	Belt	38	12.6	4	9.3	29	14.3	5	8.9	
	Rings	23	7.6	1	2.3	14	6.9	8	14.3	
	Powder	49	16.2	8	18.6	33	16.3	8	14.3	
	Extenders	11	3.6	2	4.7	6	3.0	3	5.4	
Hygiene										
	Neutral pH soap	176	58.3	20	46.5	122	60.1	34	60.7	0.640
	Acid pH soap	81	26.8	15	34.9	54	26.6	12	21.4	
	Basic pH soap	19	3.3	1	2.3	8	3.9	1	1.7	
	Non-woven gauze	145	48.0	27	62.8	93	45.8	25	44.6	
	Wet wipes	54	17.9	11	25.6	33	16.3	10	17.9	

Note: ^¥^ Benjamini–Hochberg adjustment for the inferential comparisons. Me = median; IQR = interquartile range.

**Table 3:** Poisson regression for temporal trends in PSCs.

Predictor	Coefficient	Standard Error	z-Value	Incidence Rate Ratio (IRR)	*p*
Intercept	0.031	0.075	0.412	1.031	0.681
Time (T1)	0.217	0.085	2.553	1.243	0.011
Time (T2)	0.312	0.089	3.506	1.366	<0.001
Time (T3)	0.201	0.082	2.451	1.223	0.014
Path (Green)	−0.453	0.104	−4.356	0.636	<0.001
Path (Yellow)	−0.112	0.098	−1.143	0.894	0.253
Sex (Male)	0.089	0.067	1.331	1.093	0.183
Age	0.021	0.005	4.200	1.021	<0.001
BMI (Overweight)	0.157	0.072	2.181	1.170	0.029
BMI (Obese)	0.251	0.085	2.953	1.285	0.003

Note: Goodness-of-fit statistics indicated that the Poisson regression model fits the data well (deviance = 38.2, *p* = 0.642; Pearson χ^2^ = 40.1, *p* = 0.718).

## Data Availability

The data used in this study are available upon reasonable request to the corresponding author, subject to any ethical and legal constraints.

## Public Involvement Statement

No public involvement in any aspect of this research.

## Guidelines and Standards Statement

This manuscript was drafted against the STROBE checklist for cohort studies.

## Abbreviations

The following abbreviations are used in this manuscript:

PSC	Peristomal skin complication
HRQoL	Health-related quality of life
IRR	Incidence rate ratio
IQR	Interquartile range
SD	Standard deviation
T0, T1, T2, T3	Time points (baseline, 30 days, 60 days, 90 days)
BMI	Body mass index
SF-36	Short Form-36 Health Survey
BH	Benjamini–Hochberg adjustment
